# Internal ammonium excess induces ROS-mediated reactions and causes carbon scarcity in rice

**DOI:** 10.1186/s12870-020-02363-x

**Published:** 2020-04-07

**Authors:** Shunying Yang, Dongli Hao, Man Jin, Yi Li, Zengtai Liu, Yanan Huang, Tianxiang Chen, Yanhua Su

**Affiliations:** 1 State Key Laboratory of Soil and Sustainable Agriculture, Institute of Soil Science, Chinese Academy of Sciences, No. 71, East Beijing Road, Nanjing, 210008 China; 2 University of Chinese Academy of Sciences, Beijing, 100049 China

**Keywords:** Rice, NH_4_^+^ excess, ROS, GSH cycle, Carbon scarcity, Sucrose feeding

## Abstract

**Background:**

Overuse of nitrogen fertilizers is often a major practice to ensure sufficient nitrogen demand of high–yielding rice, leading to persistent NH_4_^+^ excess in the plant. However, this excessive portion of nitrogen nutrient does not correspond to further increase in grain yields. For finding out the main constraints related to this phenomenon, the performance of NH_4_^+^ excess in rice plant needs to be clearly addressed beyond the well-defined root growth adjustment. The present work isolates an acute NH_4_^+^ excess condition in rice plant from causing any measurable growth change and analyses the initial performance of such internal NH_4_^+^ excess.

**Results:**

We demonstrate that the acute internal NH_4_^+^ excess in rice plant accompanies readily with a burst of reactive oxygen species (ROS) and initiates the downstream reactions. At the headstream of carbon production, photon caption genes and the activity of primary CO_2_ fixation enzymes (Rubisco) are evidently suppressed, indicating a reduction in photosynthetic carbon income. Next, the vigorous induction of glutathione transferase (GST) genes and enzyme activities along with the rise of glutathione (GSH) production suggest the activation of GSH cycling for ROS cleavage. Third, as indicated by strong induction of glycolysis / glycogen breakdown related genes in shoots, carbohydrate metabolisms are redirected to enhance the production of energy and carbon skeletons for the cost of ROS scavenging. As the result of the development of these defensive reactions, a carbon scarcity would accumulatively occur and lead to a growth inhibition. Finally, a sucrose feeding cancels the ROS burst, restores the activity of Rubisco and alleviates the demand for the activation of GSH cycling.

**Conclusion:**

Our results demonstrate that acute NH_4_^+^ excess accompanies with a spontaneous ROS burst and causes carbon scarcity in rice plant. Therefore, under overuse of N fertilizers carbon scarcity is probably a major constraint in rice plant that limits the performance of nitrogen.

## Background

Nitrogen (N) limitation is a leading constraint to the grain yield of rice [1, 2]. Leaf N accounts for the largest N sink of rice plant, ca. 80% of which is distributed in the chloroplast and stored as Ribulose–1,5–bisphosphate carboxylase / oxygenases (Rubisco), the primary carbon fixation enzymes of C_3_ plants [3]. Photosynthesis is tightly correlated to leaf N content [4], and more than 80% of grain N is derived from leaves in rice [5]. Hence, insufficient leaf N storage will lead to reduction of photosynthetic carbon fixation efficiency and is therefore considered as a major limitation to biomass and grain production of cereal ecosystems [6–9].

To sustain the strong and persistent N demand for higher grain yield of rice (6.4 t ha^− 1^ or above), the average N input is normally over 180 kg ha^− 1^ in China [10]. In the high-yielding rice farming areas, the N input can even reach to 300 kg ha^− 1^ and this is particularly the case for recent super-hybrid rice cultivars that achieve as high as > 10 t ha^-1^ of grain yields [11]. In soils, applied N fertilizers (e.g. urea form accounted for the majority of current N fertilizers) are rapidly converted to ammonium with the potent reactions of ureases. In rice paddy soils, ca. 70–80% of the growth period is water flooded, causing an anaerobic environment that largely prevents the process of nitrification. Therefore, NH_4_^+^ is the major form of nitrogen available to rice plant. Thus efficient dealing with NH_4_^+^ is a most important concern in rice. However, recent results show that overuse of N fertilizers strengthens excessive NH_4_^+^ accumulation in rice plant that does not correspond to further increase in grain yields [12]. Therefore, low efficiencies of N utilization and its agronomic benefits are major problems of N overuse in rice farming. The performance of such redundant portion of NH_4_^+^ is thus a meaningful issue of investigations.

The most straightforward observation caused by high NH_4_^+^ over-supply is the strong reduction of root growth. To this respect, significant advances have been achieved centering the insightful molecular mechanisms or pathways that modulate the adjustment of root morphology. In Arabidopsis, root tip contact to high NH_4_^+^ is essential for triggering the inhibitory growth of primary roots [13]. Whereas leaf contact and accumulation of toxic NH_4_^+^ impair AUX1-mediated primary polar transport of IAA to the roots thereby inhibit the emergence of lateral roots [14]. In rice, continuous exposure for several days to high NH_4_^+^ strongly inhibits seminal root elongation then causes a reduction in plant growth [15–19]. Moreover, the mechanisms of NH_4_^+^ toxicity to plants are considered to result from the accumulative consequences of divergent frustrations such as ion imbalance, intracellular pH disturbance, carbon limitation, charge/hormone imbalance or oxidative stresses [20–24]. In addition, the analysis of Arabidopsis *hsn*/*vtc* mutants indicates that GDP–mannose pyrophosphorylases-mediated protein N-glycosylation can also participate in the modulation of root elongation under NH_4_^+^ stresses [25–27]. Moreover, phytohormone signals are reported to interact with NH_4_^+^ supply and regulate plant metabolism, growth and development [13, 14, 24, 25, 27, 28]. In addition, a number of transcriptome analyses speculate that the redirections of carbohydrate metabolisms, amino acid metabolisms [19, 29] in rice plant are responsible for the toxicity of excessive NH_4_^+^. To the opposite direction, efforts have also been put to the retrieval of plant from severe stress of NH_4_^+^ toxicity. For instance, the application of gamma-aminobutyric acid (GABA) alleviates NH_4_^+^ toxicity through reducing NH_4_^+^ accumulation and assimilation capacity for a energy saving [30]. OsPTR6 promotes rice root growth by enhancing OsAMT1 expression and GS activity but at the expense of decreasing nitrogen use efficiency [31]; OsSE5 that encodes the heme-heme oxygenase 1 dedicates to relieving NH_4_^+^ toxicity by reinforcing antioxidant defense system [18].

In general, the current knowledge on plant responses to NH_4_^+^ toxicity has focused on the impacts of high NH_4_^+^ supplied outside to the roots that depends on the occurrence of a measurable phenotype to accumulate for a relatively longer time course. To this respect, ‘mixed’ influences between specific NH_4_^+^ stress responses and endogenous changes along the course of plant growth and development seem inevitable. Therefore, to one hand, the initiation of high NH_4_^+^ stress responses needs to be specifically isolated; to the other hand, the physiological and or molecular performances of NH_4_^+^ excess retained inside rice plant remain to be addressed independently of a root phenotype.

The previous study implies that the adjustment of carbohydrate metabolisms could be a notable feature in responding NH_4_^+^ status in rice in a short time period [29]. Environmental stress stimuli such as salinity or drought stresses induce the overproduction of reactive oxygen species (ROS) and promptly trigger oxidative defense responses [32, 33]. As the result, the reduction of photosynthetic CO_2_ fixation efficiency and redirection of carbohydrate metabolism could speculatively be major causes leading to compromised carbon gain and growth retardance [32, 34]. Therefore, findings or speculations from classical stress responses provide useful links to uncover the nature of the toxicity of internal NH_4_^+^ excess that has not been clearly demonstrated.

Based on the above descriptions, the present work aims at isolating the initial reactions and (molecular-) physiological responses of rice plant to the internal NH_4_^+^ excess stress before the formation of a visible phenotype. For this purpose, an acute method is established allowing drastic NH_4_^+^ excess within several hours. This is anticipated a problem-solving orientated work that could be practically useful for further understanding the performance of excess NH_4_^+^ in rice plant caused by overuse of N fertilizers. By the integration of physiological observation, transcriptomic gene expression analysis and enzyme activity assays, we demonstrate that the activation of the toxic effects of acute NH_4_^+^ excess is readily initiated by the bursts of reactive oxygen species (ROS) and subsequently leads to damages to the photosynthetic components and causes the headstream reduction of the activity of primary CO_2_ fixation. Meanwhile, elevated ROS in the plant activates GSH cycles for active radical scavenging that requires the redirection of carbohydrate metabolisms for engergization and for providing of carbon skeletons. These downstream reactions strengthen the carbon scarcity in rice plant. Finally, a sucrose feeding effectively alleviates ROS-induced frustrations, supporting that the carbon scarcity is a major constraint of rice plant in dealing with internal NH_4_^+^ excess.

## Results

### Growth inhibition under high NH_4_^+^ correlates to an NH_4_^+^ excess induced ROS burst in rice seedlings

**Fig. 1:**
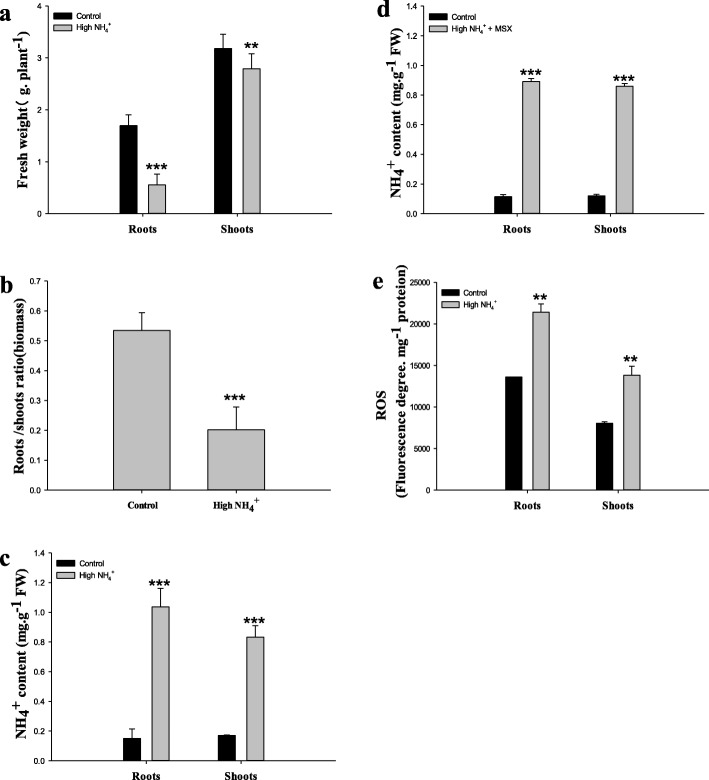
Biological and physiological analyses of NH_4_^+^ excess responses of rice. Rice seedlings aged 7 d were subjected to NH_4_^+^ treatments for 14 d (**a**-**c**, **e**). **a** Fresh biomasses of roots and shoots. **b** Root-shoot ratios. **c** Free NH_4_^+^ content and **e** Total ROS content in response to NH_4_^+^ treatments. **d** Acute NH_4_^+^ excess simulation by treating with high NH_4_^+^ for 4 h in the presence of 1 mM MSX. Rice seedlings used for this experiment were 10 d old. Values indicated means ± SE of three independent replicates. ** and *** represented statistical significances at p<0.01 and 0.001, respectively

Under persistent treatment with high NH_4_^+^ (20 mM) for 14d, a significant growth inhibition was observed compared to the control condition (1 mM NH_4_^+^) (Fig. 1a). The inhibition was more profound in roots showing a biomass reduction of up to 67% (Fig. 1a) and the root/shoot ratio was significantly lowered from approximately 0.5 down to 0.2 (Fig. 1b). Meanwhile, 7 and 5 folds higher concentrations of free NH_4_^+^ were measured in roots and shoots, respectively (Fig. 1c). Nevertheless, the strong inhibition of root growth under high NH_4_^+^ supplement was a well-defined issue that had attracted numerous investigations. Efforts have be extensively made on the elucidation of molecular mechanisms involved in root architecture adjustments in response to the accumulation of relatively long-term (several days or longer) stress effects impended by high NH_4_^+^ treatments. Here to reveal early responsive reactions that could be the trigger of the accumulative responses (growth modifications), a prompt status of internal NH_4_^+^ excess is necessarily to be established without causing visible changes in plant growth (especially roots). Therefore, L–methionine–D,L–sulfoximine (MSX), a potent inhibitor of the primary NH_4_^+^ assimilation pathway mediated by the activity of glutamine synthetases [35] was applied (1 mM) for 4 h in the presence of high NH_4_^+^ (20 mM). Considering the strong toxicity of MSX, proper conditions for the use of the drug were pre-tested to avoid lethal effects that lead to apoptotic lyses of cell components. In our hydroponics a 4 h incubation with 1 mM MSX could efficiently result in an acute NH_4_^+^ excess in both roots and shoots 5–6 fold that of the control conditions without any visible damage to rice seedlings (Fig. 1d). Thus the method allowed to simulate as fast as within 4 h, ‘saturable’ NH_4_^+^ excess circumstances inside both the roots and shoots to similar levels of the long term treatments (compare Fig. 1 c & d).

In line with the accumulation of free NH_4_^+^, bursts of reactive oxygen species (ROS) were observed (Fig. 1e), implying possible occurrence of ROS-induced reactions triggered by internal NH_4_^+^ excess.

To further demonstrate the involvement of radical species in the early response to NH_4_^+^ excess, we carried out respectively DAB (3,3′-diaminobenzidine) and NBT (nitroblue tetrazolium) histochemical staining to trace the occurrence of H_2_O_2_ and O_2_^−^ in newly-born roots and the 2nd leaves of the above treated rice plants. Results showed that upon the acute exposure to high NH_4_^+^, significant accumulation of H_2_O_2_ in both leaves and roots was detected with strong colored staining (Additional file 1, Fig. S1, a & b). The stains were readily faded to close to the control levels following a feeding of 1% sucrose (Additional file 1, Fig. S1, a & b), indicating the fallback of the H_2_O_2_ burst to the normal levels. Consistent with the observation of H_2_O_2_, the NBT stained O_2_^−^ showed closely similar changes (Additional file 2, Fig. S2, a & b). This set of data rose questions that the burst of ROS (probably independent of their composition species) was an initiation step of the toxicity mediated by NH_4_^+^ excess. Consequently, a set of ROS–triggered reactions or responses would be expected to take place as extensively described for abiotic stress responses. Indeed, according to the measurements of free amino acid contents (Additional file 3, Fig. S3), high NH_4_^+^ also caused a significant accumulation of free amino acids in both roots and leaves, resembling a common protective response of that of a drought or salinity stress.

### RNA-Seq analysis for preliminary identification of genes modulated by NH_4_^+^ excess

According to above description, rice seedlings were treated with high NH_4_^+^ in the presence of 1 mM MSX for 4 h to establish an internal environment of NH_4_^+^ excess. Then RNA-Seq analyses were carried out to seek for molecular responses related to this circumstance. Respectively 1077 and 1040 differentially expressed genes (DEGs) were obtained from roots and shoots, with > 2 fold changes in their transcriptional levels (Additional file 4). Based on the GO classification, these genes mainly belonged to “metabolic process”, “molecular function”, “binding” and “biological process” (Additional file 5). Further KEGG pathway analysis revealed possible involvements of the responsive genes (DEGs) in stress response, photosynthetic adjustment, carbohydrate and amino acid metabolisms, preparation of hormone signaling pathways and re-adjustment of NH_4_^+^ transport (Additional file 6). The significantly regulated genes were further summarized below within the framework of major processes they participate.

### Activation of GSH cycle for ROS scavenging

**Fig. 2:**
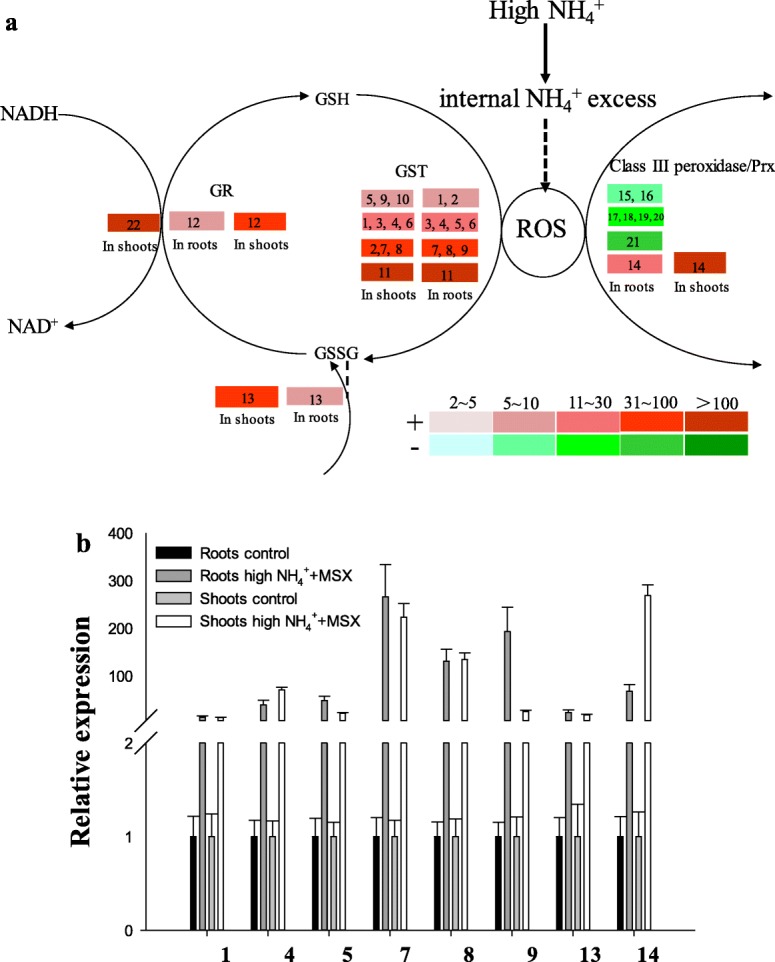
Gene expression analyses of responsive ROS scavenging genes. **a** Differentially expressed genes obtained by RNA-Seq were illustrated in relation to the major pathways they were involved. Colored columns corresponded to fold changes of the transcripts as indicated by the definition of color gradients (bottom). The symbol ‘+’ and the red gradient columns represented up-regulated genes and the fold of induction; while ‘-‘and the green gradient columns referred to down-regulated genes. **b** The qRT-PCR validation of randomly selected genes coding for ROS scavenging systems. The relative expression levels were normalized against *OsActin.* Values indicated were means of three independent replicates. Numbered responsive genes were annotated as followings: 1. Os01g0949700, putative glutathione S-transferase; 2. Os03g0785900, probable glutathione S-transferase GSTU1; 3. Os01g0369700, putative glutathione transferase 4; 4. Os01g0949800, putative glutathione S-transferase; 5. Os01g0949750, putative glutathione S-transferase; 6. Os10g0365200, glutathione S-transferase; 7. Os10g0527800, glutathione S-transferase OsGSTU12; 8. Os10g0481300, glutathione S-transferase; 9. Os10g0525500, glutathione S-transferase parC; 10. Os01g0372400, putative glutathione S-transferase; 11. Os10g0528300, glutathione S-transferase OsGSTU4; 12. Os10g0415300, glutathione reductase; 13. Os08g0557600, monodehydroascorbate reductase; 14. Os07g0638400, 1-Cys peroxiredoxin B; 15. Os05g0499300, peroxidase 1; 16. Os07g0677300, peroxidase 2; 17. Os05g0134800, Class III peroxidase 67; 18. Os02g0236600, Class III peroxidase 27; 19. Os03g0234900, Class III peroxidase 39; 20. Os03g0368000, Class III peroxidase 42; 21. Os06g0695300, Class III peroxidase 92; 22. Os07g0564500, NADH dehydrogenase [EC:1.6.99.3]

Following the acute NH_4_^+^ excess and the bursts of ROS (Fig. 1c, d, e), a most remarkable response was the strong induction of glutathione S–transferases (GST) genes (Fig. 2). Eleven GST genes were typically upregulated for > 7 or even some tens to hundreds fold both in roots and shoots (Fig. 2ab, genes#1–11). Among those GSTs, a OsGSTU4 (Os10g0528300, Fig. 2a, gene#11) was the most severely induced by > 300 and > 600 fold in roots and shoots respectively, followed by 2 putative GST genes (Os10g0481300 and Os10g0527800) that were upregulated by 50–100 fold in both parts. Whereas Os10g0525500 (77 fold) and Os03g0785900 (90 fold) showed strong induction in roots and shoots respectively (Fig. 2a, b). Since GSTs catalyze the transfer of superoxide free radicals to reductive glutathione (GSH) that leads to the detoxification of the oxidants, these changes in GST gene expression provide indications for the critical involvement of the GSH cycle in scavenging the NH_4_^+^ excess induced ROS.

In line with strengthened demand of reducing power, a putative glutathione reductase gene (Os10g0415300) responsible for the recruitment of GSH was moderately upregulated (~ 8 fold) in roots and vigorously enhanced by 70 fold in shoots (Fig. 2a). Meanwhile, a NADH dehydrogenase gene (Os07g0564500) was stimulated by 127 folds in shoots, partly reflecting the coupling of energization and reducing power with the operation of the GSH cycle (Fig. 2a).

In addition to profound changes related to the GSH cycle, 7 peroxidase genes were suppressed in roots whereas a putative 1-Cys peroxiredoxin B gene (Os07g0638400) was significantly induced in both roots (19 fold) and shoots (179 fold) (Fig. 2a), corresponding to the contradictory roles of peroxidases in the cleavage / homeostasis maintenance of ROS [36].

### Suppression of photosynthesis components and contrasting regulation of energy producing carbohydrate metabolism

**Fig. 3:**
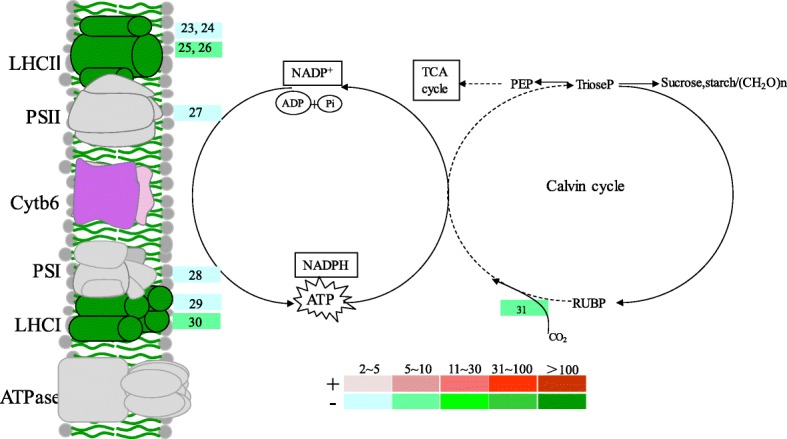
Responsive genes involved in photosynthesis. Differentially expressed genes obtained by RNA-Seq were illustrated in relation to the major processes they were involved. Colored columns corresponded to fold changes of the transcripts as indicated by the definition of color gradients (bottom). The symbol ‘+’ and the red gradient columns represented up-regulated genes and the fold of induction; while ‘-‘and the green gradient columns referred to down-regulated genes. Numbered responsive genes were annotated as followings: 23. Os03g0592500, light-harvesting complex II chlorophyll a/b binding protein 2 (LHCB2); 24. Os07g0558400, light-harvesting complex II chlorophyll a/b binding protein 4(LHCB4); 25. Os01g0720500, light-harvesting complex II chlorophyll a/b binding protein 1 (LHCB1); 26. Os09g0346500, light-harvesting complex II chlorophyll a/b binding protein 1 (LHCB1); 27. Os03g0333400, photosystem II Psb27 protein (psb27); 28. (Os08g0560900), photosystem I subunit II (psaD); 29. Os06g0320500, light-harvesting complex I chlorophyll a/b binding protein 1 (LHCA1); 30. Os02g0197600, light-harvesting complex I chlorophyll a/b binding protein 3 (LHCA3); 31. Os12g0292400, ribulose-bisphosphate carboxylase small chain [EC:4.1.1.39] (rbcS)

The chlorophyll a/b binding proteins of light-harvesting complexes (LHCs), also known as antenna proteins, are involved in gathering light energy (photons) of the primary reaction of photosynthesis [37]. Then trapped photons and electrons are transported to reaction center for further photochemical reactions. Disruption of these processes by photodamage, herbicides, or accumulation of highly active radicals will obviously hinder the progress of photosynthesis. Upon a prompt (4 h) NH_4_^+^ excess treatment, 6 genes coding for the LHC antenna proteins (4 LHC II and 2 LHC I, respectively), a PS I and a PS II reaction center genes were almost evenly suppressed by approximately 5 fold (Fig. 3), indicating the onset of the reduction of efficiencies of photon gathering and transfer. It would be easily supposed that apparent suppression of photosynthesis would accumulate along the progress of NH_4_^+^ excess stress and growth inhibition would consequently occur. Meanwhile, Os12G0292400 coding for the small chain of Rubisco, the key enzyme catalyzes the fixation / assimilation of CO_2_, was downregulated by ~ 5 fold (Fig. 3), providing further indication of compromised photosynthetic carbon production. Therefore, plant NH_4_^+^ excess initiates and probably also develops the disruption of photosynthesis by interfering in the primary reaction and the Calvin Cycle.

**Fig. 4:**
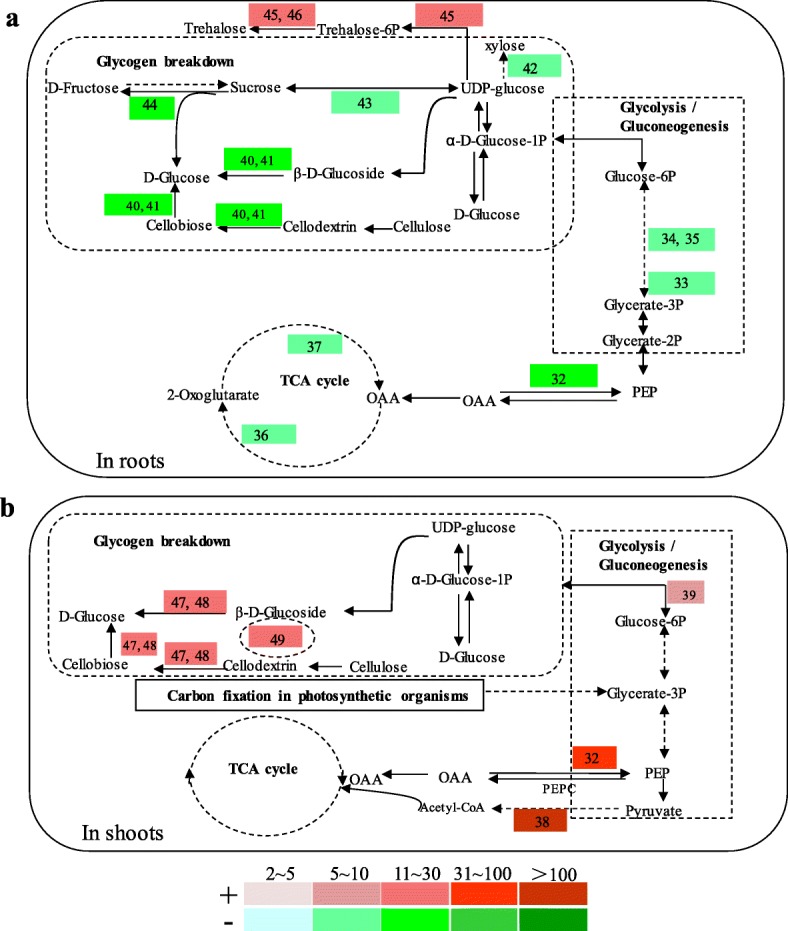
Responsive genes involved in carbohydrate metabolisms in roots (**a**) and shoots (**b**). Differentially expressed genes obtained by RNA-Seq were illustrated in relation to the major processes they were involved. Colored columns corresponded to fold changes of the transcripts as indicated by the definition of color gradients (bottom). The symbol ‘+’ and the red gradient columns represented up-regulated genes and the fold of induction; while‘-’ and the green gradient columns referred to down-regulated genes. Numbered responsive genes were annotated as followings: 32. Os10g0204400, phosphoenolpyruvate carboxykinase (ATP) [EC:4.1.1.49] (pckA); 33. Os05g0482700, 2,3-bisphosphoglycerate-independent phosphoglycerate mutase [EC:5.4.2.12] (gpmI); 34. Os08g0120600, fructose-bisphosphate aldolase, class I [EC:4.1.2.13] (ALDO); 35. Os01g0905800, fructose-bisphosphate aldolase, class I [EC:4.1.2.13] (ALDO); 36. Os05g0573200, isocitrate dehydrogenase [EC:1.1.1.42] (IDH); 37. Os05g0574400, malate dehydrogenase [EC:1.1.1.37] (MDH2); 38. Os05g0469600, pyruvate decarboxylase [EC:4.1.1.1]; 39. Os02g0600400, glucose-6-phosphate 1-dehydrogenase [EC:1.1.1.49] (G6PD); 40. Os09g0491100, beta-glucosidase [EC:3.2.1.21]; 41. Os02g0131400, beta-glucosidase [EC:3.2.1.21]; 42. Os04g0640700, beta-D-xylosidase 4 [EC:3.2.1.37] (XYL4); 43. Os03g0401300, sucrose synthase [EC:2.4.1.13]; 44. Os02g0106100, beta-fructofuranosidase [EC:3.2.1.26] (sacA); 45. Os08g0445700, trehalose 6-phosphate synthase / phosphatase [EC:2.4.1.15 3.1.3.12] (TPS); 46. Os06g0222100, trehalose 6-phosphate phosphatase [EC:3.1.3.12] (otsB); 47. Os05g0366600, beta-glucosidase [EC:3.2.1.21]; 48. Os09g0511600, beta-glucosidase [EC:3.2.1.21], 49. Os05g0438500, inorganic pyrophosphatase [EC:3.6.1.1]

Radical scavenging enzymes are activated and energized by the ATP producing processes including glycolysis and the TCA pathways. However, several genes involved in glycolysis and the TCA cycle were contrastingly regulated in roots and shoots (Fig. 4). In roots, genes coding for 2,3-bisphosphoglycerate-independent phosphoglycerate mutase (Os05g0482700, gene#33) and fructose-bisphosphate aldolases (Os08g0120600, gene#34 and Os01g0905800, gene#35) of glycolysis, isocitrate dehydrogenase (Os05g0573200, gene#36) and malate dehydrogenase (Os05g0574400, gene#37) of the TCA cycle were down-regulated by 6–10 fold following 4 h of NH_4_^+^ excess treatments (Fig. 4a). Meanwhile genes involved in glycogen breakdown were suppressed in roots (Fig. 4a): phosphoenolpyruvate carboxykinase (Os10g0204400, gene#32, − 19 fold), beta-glucosidase (Os09g0491100, gene# 40, − 11 fold), beta-glucosidase (Os02g0131400, foldgene#41,–15 fold), beta-D-xylosidase 4 (Os04g0640700, gene#42, −7fold), sucrose synthase (Os03g0401300, gene#43, −8fold), beta-fructofuranosidase (Os02g0106100, gene#44, − 11 fold). To the contrary, enhanced glycolysis/glycogen breakdown in shoots could be indicated by the upregulation of related genes (Fig. 4b): glucose-6-phosphate 1-dehydrogenase (Os02g0600400, gene#39, + 5 fold), inorganic pyrophosphatase (Os05g0438500, gene#49, + 18 fold), phosphoenolpyruvate carboxykinase (Os10g0204400, gene#32, + 34 fold), beta-glucosidase (Os05g0366600, gene#47, + 12 fold), beta-glucosidase (Os09g0511600, gene#48, + 20 fold). Notably, a pyruvate decarboxylase gene (Os05g0469600, gene #38) of glycolysis, was specifically induced in shoots (Fig. 4b). In addition, two genes Os06g0222100 and Os08g0445700 coding for trehalose 6-phosphate synthase/phosphatases were induced by respectively 15 and 13 fold in roots (Fig. 4a, genes #45,46), suggesting enhanced biosynthesis of the ‘survival substance’ [32] trehalose induced by NH_4_^+^ excess stress.

### Sucrose feeding alleviates NH_4_^+^ excess stress responses

**Fig. 5:**
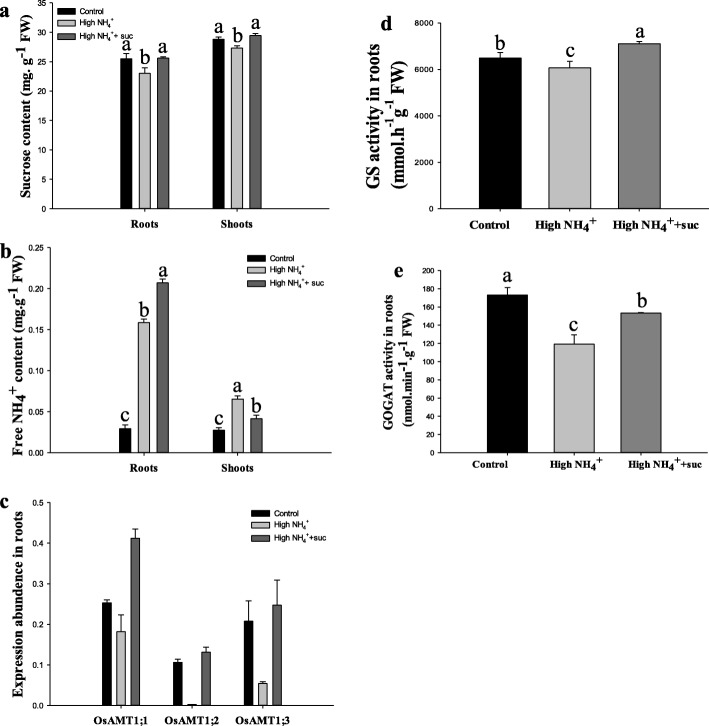
Effects of sucrose feeding on NH_4_^+^ accumulation, uptake and assimilation processes. Seedlings aged 10 d were subjected to control (1 mM NH_4_^+^), high NH_4_^+^ (20 mM) or high NH_4_^+^+ suc (20 mM NH_4_^+^ + 1% sucrose) treatments for 24 h. **a** Sucrose content, **b** free NH_4_^+^ content, **c** the expression profiles of OsAMT1;1, OsAMT1;2 and OsAMT1;3 determined by qRT-PCR, **d** GS enzyme activity, **e** GOGAT enzyme activity. Data were means ± SE of three independent replicates. Letters on the bars represented statistical significances

The above analyses revealed rather frustrating responses to NH_4_^+^ excess stress in rice plant that closely associated with the consumption of carbohydrates for energy demand. Hence a sugar scarcity could accumulatively (to a longer time course) result in growth inhibition. To test this hypothesis, we fed 1% of sucrose as a sugar compensation to the high NH_4_^+^ (20 mM) hydroponics for 24 h. This treatment compensated the sucrose consumption at high NH_4_^+^ and allowed the sucrose contents in roots and shoots to restore to equivalent levels of the control (1 mM NH_4_^+^) conditions (Fig. 5a). The sucrose feeding treatments further increased the free NH_4_^+^ contents in roots, but significantly reduced NH_4_^+^ accumulation to the shoots (Fig. 5b).

Under high NH_4_^+^ conditions, the expression levels of 3 AMT1 genes (*OsAMT1;1*–Os04g0509600, *OsAMT1;2*–Os02G0620500 and *OsAMT1;3*–Os02G0620600) were suppressed respectively by 3, 67 and 6 fold in roots, implying a reduction in NH_4_^+^uptake activity. With the supplement of sucrose (1%) to the high NH_4_^+^ hydroponics (Fig. 5c), their expression levels restored to close to the ‘normal’ levels (at 1 mM NH_4_^+^).. This implied a release of ammonium transporting activity from suppression by NH_4_^+^excess, thus contributed to enhanced NH_4_^+^ accumulation in roots under high NH_4_^+^ plus sucrose condition. Whereas the reduced free NH_4_^+^ content under the same condition in shoots indicated probably the efficient utilization of NH_4_^+^ upon the addition of sucrose (Fig. 5b). Meanwhile the GS (Fig. 5d) and GOGAT (Fig. 5e) activities were respectively enhanced by 17% (GS) and 29% (GOGAT) in roots following the sucrose feeding treatments, indicating a restoration of NH_4_^+^ assimilation activities from initial suppression by NH_4_^+^ excess.

**Fig. 6:**
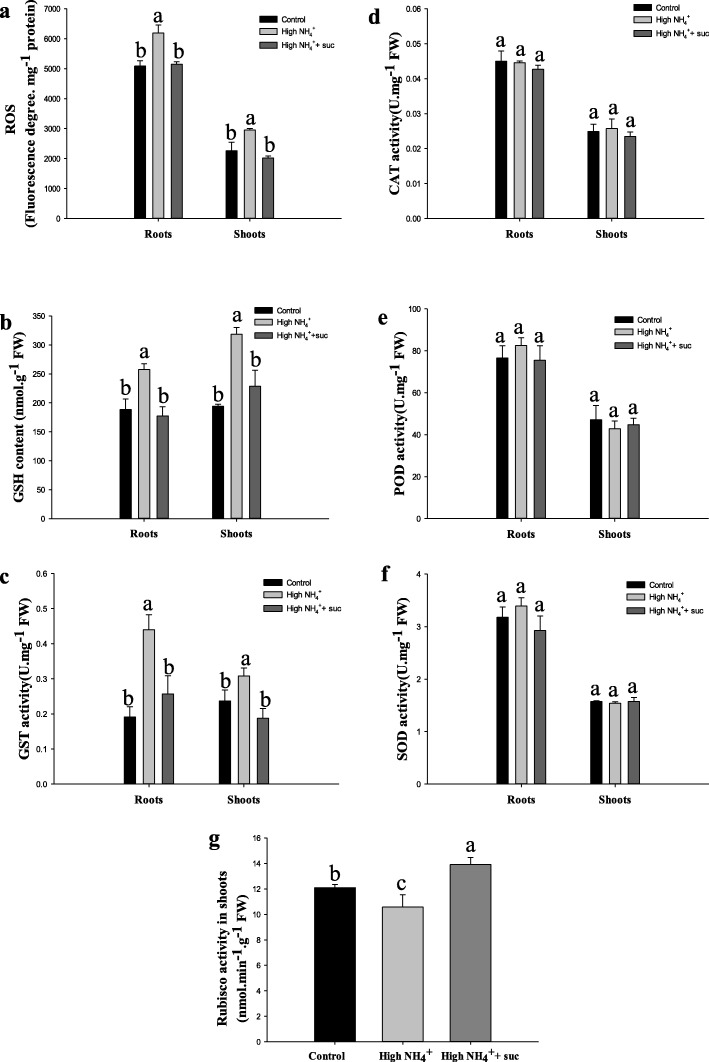
Effects of sucrose feeding on ROS accumulation, ROS scavenging enzymes and Rubisco activities. Seedlings aged 14 d were subjected to control (1 mM NH_4_^+^), high NH_4_^+^ (20 mM) or high NH_4_^+^+ suc (20 mM NH_4_^+^ + 1% sucrose) treatments for 24 h. **a** Total ROS accumulation represented by fluorescence degrees, **b** GSH content, **c**-**g** Enzymes activity assays for **c** GST, **d** CAT, **e** POD, **f** SOD and **g** Rubisco. Experimental conditions were the same as described in Fig. 5. Data were expressed as means ± SE of three independent replicates. Letters on the bars represented statistical significances

Upon the compensation of sucrose source, the total ROS contents in both roots and shoots were lowered down by 20–30%, close to the levels determined at control (1 mM NH_4_^+^) conditions (Fig. 6a). Accordingly, the GSH content and GST activity were significantly reduced to the initial levels (at 1 mM NH_4_^+^), no longer showing strong induction by NH_4_^+^ excess (Fig. 6b, c). Unexpectedly, no significant changes were observed with the activities of classical defense enzymes CAT, POD and SOD under either treatment (Fig. 6d, e, f). Together with the gene expression analyses (Fig. 2), our results demonstrated that the activation of GSH reducing pathway is probably a featured response of rice in dealing with NH_4_^+^ excess and ROS accumulation. Finally, in consistent with the decreased level of ROS, Rubisco activity was elevated by 24% (compared with high NH_4_^+^) in shoots with the presence of sucrose feeding (Fig. 6g), suggesting enhanced efficiency of primary CO_2_ fixation activity.

Taken together, this set of experiments indicated that sucrose feeding could effectively alleviate rice plant from carbon scarcities exerted by internal NH_4_^+^ excess and ROS stresses.

## Discussion

### The performance of internal NH_4_^+^ excess is an issue of physiological and practical significance

Due to particular water-flooding and anaerobic environment, NH_4_^+^ retains as the major form of N nutrient available to paddy rice. For the present high-yielding rice production in China, to satisfy the strengthened N demand for high levels of grain yields, nitrogen fertilizers are applied as high as typically 300 kg ha^-1^ N. The total amount of nitrogen normally composes of ~ 40% of basal N at the seedling stage and respectively 30% of topdressed N at the tillering and grain-filling stages to ensure sufficiently higher N contents in the roots and photosynthetic leaves. This amount of nitrogen is thought to be overused since the grain yield is saturated; but is necessary for farms’ goal of a high grain yield [10–12, 38, 39]. As the consequence of N overuse, a persistent internal NH_4_^+^ excess circumstance exists for rice plant to cope with. Therefore, a study focuses on such farming-intervened special circumstances would be helpful in discovering ‘bottlenecked’ constraints and adaptation strategies related to the (molecular) physiological and agronomic respects of N performances in rice. Then questions rise for researchers: what the excessive portion of N does in rice plant? What is the main constraint that limits the excess N from conversion to further productivity?

Under the field conditions of N overuse (say 300 kg ha^-1^ N), suppose the N fertilizer exists totally in the form of NH_4_^+^ ions and are mainly distributed within a depth of 30 cm (including water body), the 4:3:3 proportions of total N (NH_4_^+^) will roughly result in 2.2, 1.7 and 1.7 mM of NH_4_^+^ concentrations, such concentrations will be further compromised due to the buffering capacity of the soil (NH_4_^+^ adhered to soil particles), finally the free NH_4_^+^ ions around the roots could be in a “safe” range that does not stress the roots. As the result, N overuse in rice field is not exactly an external high NH_4_^+^ stress, for N fertilizer topdressing is normally applied almost evenly to the surface of water flooded field, not by localized dressing that brings high NH_4_^+^ to the roots. In this circumstance, the problem of N overuse can be simplified as an NH_4_^+^ excess inside the plant.

It is to this respect we design the experiments. Although like many others, for better controlling of experimental conditions we use hydroponics to address the question. Particular attentions have been paid to avoid growth divergence during the whole set of experiments. To isolate the specific reactions initiated by internal NH_4_^+^ excess, we establish an acute method that creates sufficient levels of NH_4_^+^ excess in 4 h by the presence of MSX to block (targeted to GS) the assimilation of NH_4_^+^ acquired into the plant. For the use of MSX, we clearly notice its strong toxicity to plants and serious precautions are made to find a “safe” condition by pre-testing its effects on induction of significant NH_4_^+^ accumulation in shoots and its toxic influences to the seedlings. When MSX was supplied at 0.1 mM concentration, NH_4_^+^ accumulation in shoots within 4 h is much less profound (1–2 fold) than at 1 mM (5–6 fold). Within the first 4 h of incubation with 1 mM of MSX, rice seedlings remain clearly unaffected, indicating no injury occurred at this time period; with extended incubation to 12 h, leaves turn yellowish and somehow curl, and finally up to 24 h, seedlings start dying. In addition, our previous work on optional gene expression observation [29], we finally use 4 h treatments with high NH_4_^+^ and the presence of 1 mM MSX.

### The toxicity of internal NH_4_^+^ excess initiates with bursts of ROS

High NH_4_^+^ stress and toxicity have been considered as a major human-intervened environmental distress exerted on plants and attracted extensive research interests. Researches on these topics have focused on the identification of mechanisms or pathways that primarily modulate the biological modifications of root architectures [20–24]. Solid evidences have shown the re-configuration of plant root morphology in response to NH_4_^+^ stresses is tightly controlled through the interactions with plant hormone signaling pathways [13, 14, 24, 25, 27]. Whereas NH_4_^+^ toxicities could be attributed to ion imbalances [20], intracellular pH disturbance [40], energy consumption due to invalid NH_4_^+^ cycles in roots [13, 41]. Assessments of NH_4_^+^ stress responses in plant roots and its biological toxicities, to a great extent, relies on the establishment of measurable growth phenotypes that requires effects or reactions to accumulate for a desired time course. These analyses are obviously important in addressing the mode-of-action of physiological effects or processes developed along the progresses of the treatments. To the other hand, since plants keep growing and developing during the experimental periods, these accumulative observations might be not satisfactory for capturing the initial reactions or the nature of NH_4_^+^ excess stresses. Therefore, it seems inevitably necessary to isolate the initial reactions triggered by internal NH_4_^+^ excess from rather mixed accumulative effects.

Our results with physiological measurements and histochemical observations clearly demonstrate that the burst of ROS radicals is a foremost straightforward consequence readily along the acute internal NH_4_^+^ excess (Fig. 1c- e; Additional files 1, 2). Then we obtain a whole set of indications supporting that the essential nature of the toxicity of NH_4_^+^ excess in rice plant is probably initiated by the induction of ROS bursts and the downstream reactions: 1) since photosynthetic components are sensitive to radical injuries, we observe reduced abundances of genes involved in photon-caption and compromised primary CO_2_ fixation activity of the Rubisco enzymes (Figs. 3 and 6g); 2) downstream the ROS burst, we identify that GSH cycling acts as a seemingly specific defense mechanism for scavenging ROS, using both transcription and enzyme activity changes of GST genes as indicators (Figs. 2 and 6); 3) to energize the highly energy-consuming ROS cleavage reactions, we observe gene expression indications for a reduced complex sugar synthesis and strongly enhanced breakdown of active simple sugars in shoots (Fig. 4), such shift in carbon metabolism points to a strengthened production of carbon skeletons. Conversely, the enhancements of energy and carbon skeleton production seem not accordingly take place in roots (Fig. 4), implying a sacrifice of root growth for stress escaping.

To this end, the nature of the toxicity of NH_4_^+^ excess in rice plant resembles largely that has been well defined for an abiotic stress, such as drought or salinity stresses described in other plant species [32, 34], and shares same origins—oxidative injuries and ROS induced energy and carbon skeleton consumption. There are sufficient speculations of the induction of ROS under high NH_4_^+^ stresses. In tobacco and grapevine suspension cells, a 24-treatment with high NH_4_^+^ or NaCl results in the generation of ROS that signals the redirection of amino acid synthesis and metabolisms [42] (Skopelitis et al., 2006 PC). In the hydroponics of *Myriophyllum mattogrossense*, the induction of oxidative stress responses by an excess of ammonia (NH_3_ and NH_4_^+^) is deduced from the increased activities of antioxidative protection enzymes [43] (Nimptsch et al., 2007, Chemosphere). After a 26 h of N depletion, resupplement of even 1 mM of NH_4_^+^ to *Arabidosis* is suspected to induce oxidative stress responses as deduced from elevated activities of antioxidant scavengers [44] (Patterson 2010 PEC). Under 25 mM NH_4_^+^, elevated H2O2 can be detected in *Arabidosis* and participates the modulation of AMOS1/EGY1-dependent ABA signaling 28]. In rice, continuous exposure for 6 days to extremely high concentration of NH_4_^+^ (80 mM) results in significant accumulation of ROS and activates the involvement of heme oxygenase 1 in the alleviation of NH_4_^+^ toxicity [18] (Xie et al., 2015 PCE). In general, for most of phenotype-related assessments, plants are subjected to continuous NH_4_^+^ stresses so that solid phenotypes develop, and in these cases, elevated of ROS levels are considered as fundamental signals or triggers for the activation of specific molecular pathways. For the identification of “early” responses before the formation of a growth phenotype, transcriptomic studies focus on the gene expression regulation to form speculations on the involvement of ROS and downstream reactions in plants’ responses to high NH_4_^+^ stresses [19, 29]. However, the induction of ROS remains to be clearly quantified in relation to the accumulation of free NH_4_^+^ inside the plant and the internal effects of NH_4_^+^ excess also need to be isolated from stress responses impended exogenously to the roots. Here in this report, we present with physiological and histochemical observations, the acute induction of ROS readily accompanied with the formation of internal NH_4_^+^ excess, providing a solid evidence that the frustrating performance of internal NH_4_^+^ excess integrates and probably also originated from the burst of ROS.

*Carbon scarcity is a major constraint on the effectiveness of the internal NH*_*4*_^*+*^ *excess*

Our whole set of data supports a prediction that a carbon scarcity occurs along with the internal NH_4_^+^ excess, including the headstream reduction of photosynthetic carbon assimilation (Fig. 3) and the redirection of carbohydrate metabolisms to enhanced energy and C skeleton production (Fig. 4). The prediction is further proofed by the sucrose feeding experiments that eventually cancel the negative effects associated with NH_4_^+^ excess (Fig. 5 and 6). Sucrose is chosen for the complementation of carbon scarcity because this sugar is the major form of active carbon source in the phloem and can be transported and allocated in plant tissues and organs [45].

However, the issue of “carbon scarcity” is rather a common view as the consequence of a stress response, since numerous reports have raised such speculation [20–24, 29, 32, 46]. In this report, we present data sets that point to the initiation and development of carbon scarcity (Figs. 2, 3 and 4). Therefore, the occurrence of carbon scarcity and its relationship to NH_4_^+^ excess and / or N overuse are no longer a hypothesis but a conclusive notion with solid data supports. To this respect, the nature of NH_4_^+^ toxicity can be explained as the development and accumulation of carbon scarcity that eventually lead to growth inhibition or death of the plants.

As demonstrated by sucrose feeding experiment of the present work (Figs. 5 and 6) and reports from Arabidopsis under high NH_4_^+^ [46], salinity stresses [47] (Qiu et al., Biologia Plantarum) or chickpea under salinity stresses [48] (Khan et al., 2016 JXB), enhancement of active sugar income would be an efficient approach of overcoming the shortage of carbon source. Whereas in the field, supplement of elevated concentration of CO_2_ is a straightforward regulation to enhance photosynthetic carbon production and the grain yields of cereals [49–52] (Ainsworth 2005; Leakey 2009; Becker 2016; Kimball 2016 Current opinion). However, reports have shown that continuous exposure to high CO_2_ for longer than 3–5 growth seasons leads to a phenomenon of acclimation due to a significant reduction of stomatal conductance and limits of nitrogen nutrient [49, 53] (Seneweera et al., 2002 Funct. Plant biology; Ainsworth 2005). Such acclimation to elevated CO_2_ can be partly hindered by supplement of sufficient nitrogen [54] (Stitt, 1999), providing an implication that this approach may be more effective under current N overuse in rice. Additionally, reports has shown that the addition of CaCO_3_ to the roots alleviates the growth inhibition of cucumber from high NH_4_^+^ stress [55] (Roosta, 2008). This manner of carbon feeding is expected to be practically useful for China’s rice farming because a great plot of rice production locates to the low pH red soils, and lime is often applied as a soil conditioner for the purpose of pH neutralization.

Efficient NH_4_^+^ uptake through AMT transporters is closely related to the removal of the substrate by GS-mediated assimilation processes, ineffective removal or accumulation of NH_4_^+^ would hinder the uptake of the ions—a phenomenon of so–called feedback inhibition [[46, 56–58]. The strong suppression of root-specific AMT genes, *OsAMT1;2* and *OsAMT1;3* under rapid NH_4_^+^ excess conditions (Fig. 5c) and reduction of GS and GOGAT activities (Fig. 5d, e) provide further evidence of such feedback regulation in rice. Here, upon the carbon compensation by sucrose feeding, the NH_4_^+^ assimilation activities restore to normal rates (normal NH_4_^+^, 1 mM) and the AMT expression levels are accordingly enhanced (Fig. 5c, d, e), supportedly suggesting that carbon scarcity may be a major cause that leads to feedback inhibition of NH_4_^+^ uptake.

## Conclusions

To summarize, the present work reveals that the essential nature of internal NH_4_^+^ excess stresses in rice plant is closely correlated to its accompanying ROS bursts. Elevated oxidative radicals impair the photosynthetic components and lead to reduced primary carbon production. The activation of ROS scavenging processes redirects the carbohydrate metabolisms for enhanced energy and carbon skeleton production and strengthens carbon scarcity in rice plant. A sucrose feeding effectively alleviates frustrating stress responses. Therefore we conclude that carbon scarcity is probably a major constraint on the effectiveness of internal NH_4_^+^ excess under current N fertilizer overuse of high-yielding rice.

## Methods

### Plant growth and treatments

Rice seeds of *Oryza. sativa* ssp. Japonica *Nipponbare* were obtained from Prof. Yingguo Zhu’s group, College of Life Sciences, Wuhan University. The seeds were surface sterilized, germinated and seedlings were grown in a growth chamber according to previously described [29]. The growth chamber was set with 16/8 h day/night, 27/25 °C,day/night; The light intensity was 400 μmol m^− 2^ s^− 1^, relative humidity was set at 70%. Seedlings were grown in the IRRI solution [29] until desired ages. The pH of hydroponics was buffered to 5.7 with 10 mM MES and renewed every 48 h. For treatments seedlings of uniform sizes were transferred to cylinder polyvinyl chloride culturing tanks (10 cm inner diameter and 15.5 cm height) filled with 1.0 L of nutrient solutions supplied with desired concentrations of NH_4_^+^. Eight seedlings separated into 4 holes were placed onto the lids of each culturing tanks. At the time of harvest, seedlings of each tank were pooled and served as one duplicate of every treatment. The treatments were duplicated in 3 individual culturing tanks. For long-term growth tests, uniform seedlings of 7 d were treated with either 1 mM (control) or 20 mM (high NH_4_^+^) of NH_4_Cl supplemented to nitrogen–free IRRI solutions for further 14 days with daily refreshment of the culture solutions. To achieve a rapid NH_4_^+^ excess in rice plants without causing a growth discrepancy, so that NH_4_^+^ excess-responsive genes could be analyzed at the early stages of responses, 10-d old seedlings were promptly treated with ‘control’ or ‘high NH_4_^+^’ (see above) for 4 h in the presence of 1 mM methionine sulfoximine (MSX, a potent inhibitor of glutamine synthetase) to block the major assimilation of NH_4_^+^. For sucrose feeding experiments, 14-d old seedlings were treated with control (1 mM NH_4_^+^) or high NH_4_^+^ (20 mM NH_4_^+^) in IRRI solution in the presence of 1% (w/v) sucrose for 24 h. To avoid the burst of microbes associated with sucrose–containing hydroponics, antibiotics penicillin (50 mg L^− 1^) and chloramphenicol (25 mg L^− 1^) were included to the culturing solution according to Lejay’s description [59]. Same strength of antibiotics was included in the control seedlings (control NH_4_^+^ and high NH_4_^+^ treated plants). Also in order to prevent possibly undesired impacts, the treatment was limited to within 24 h.

### RNA-Seq and quantitative real-time PCR analyses

Total RNAs from treated root or shoot samples was extracted withTRIzol total RNA extraction kit (Invitrogen, Carlsbad, CA, USA) according to the manufacturer’s protocol. For RNA–Seq analysis, RNAs from control (1 mM NH_4_^+^)or high NH_4_^+^plus 1 mM MSX treated (4 h) tissue samples were used for library construction and sequencing. Data extraction, identification of differentially expressed genes (DEGs) and functional annotation were analyzed according to our previous work [29]. DEGs were designated with expression fold changes greater than 2 (*p* < 0.05) between the rapid NH_4_^+^ accumulation (high NH_4_^+^ + MSX) and the control conditions.

Quantitative real-time PCR (qRT–PCR) analyses was carried out to reveal possible responses at the gene expression level related to special conditions such as NH_4_^+^ excess stress or sucrose feeding treatments. About 1 μg of total RNA was used to synthesize first–strand cDNAs using the PrimeScript™ RT Master Mix (Perfect Real Time, TaKaRa, Japan) according to the manufacturer’s description. Primer sequences used for qRT-PCR were listed in Additional file 7. Thermocycling and fluorescence detection were performed with C1000 Thermal Cycler CFX96 Real–Time System (Bio–Rad) using the SYBR Premix Ex Taq (TaKaRa, Japan) as indicated by the manufacturer’s protocol. The reaction was performed under the following conditions: 95 °C for 30 s, followed by 44 cycles of 95 °C for10 s, 60 °C for 15 s and 72 °C for 15 s. For fold change analysis, gene expression abundance was quantized with –2^ΔΔCt^ and normalized against the internal OsActin gene. PCR amplifications were repeated three times using cDNA templates synthesized from three independent plant samples.

### Tissue free NH_4_^+^, free amino acids, GSH and sucrose contents assays

Fresh root or shoot samples (0.2 g) were ground into fine powder in liquid N_2_ and homogenized in 5 ml of 0.3 mM sulfuric acid. The supernatant was harvested by centrifugation with 20,000 g at 4 °C for 20 min. For free NH_4_^+^measurements, aliquots of supernatant (200 μL) were mixed with 4.9 mL each of phenol-sodium nitroprusside solution and alkaline hydrochlorite solution according to the method of Weatherbur [60]. The color reaction was allowed to develop at room temperature for 1hbefore the colorimetric absorbance been measured at 625 nm. The content of free amino acids was determined by a T–free AA assay kit (Nanjing Jiancheng Bioengineering Institute, Nanjing, China) using glycine as the standard [61]. The tissue GSH content was measured according to Cheng’s method [62]. The tissue sucrose extraction was carried out according to Sonnewald’s method [63], and the tissue sucrose contents determination was measured based on Stitt’s description [63, 64].

### Determination of total ROS, histochemical staining and ROS scavenging enzyme activity assays

Total reactive oxygen species (ROS) contents induced by internal NH_4_^+^excess were assessed by 2′,7′-Dichlorofluoresceindiacetate (H_2_DCF-DA) method [65]. Fresh root or shoot samples were first made into single cell suspensions with single cell suspension medimachine after removing cell wall with cellulase and macerozyme. Then H_2_DCF–DA was added to 200 μL single cell suspensions to a final concentration of 10 μM, mixed and incubated at 37 °C for 30 min. Cells were pelleted by centrifugation at 1000 g for 10 min, washed twice with PBS, and diluted with PBS for fluorescence assay. The absorbance was determined on a fluorescence microplate reader (BioTek Instruments, Winooski, VT) at an excitation wavelength of 500 nm and an emission wavelength of 530 nm according to the descriptions of Karlsson and Sun [66, 67].

Relatively uniformed and newly-grown roots and the second leaf of rice seedlings were used for histochemical staining [68]. The generation of hydrogen peroxide (H_2_O_2_) or superoxide (O_2_^−^) in situ was detected by using 3,3′ -diaminobenzidine (DAB) or nitroblue tetrazolium (NBT) staining, respectively [68, 69]. Samples were analyzed and photographed using a fluorescence microscope (Nikon 80i). At least three leaves or roots were stained independently for these experiments.

For antioxidative enzyme activity analyses, 0.2 g of fresh root or shoot samples were ground in liquid N_2_, homogenized and crude extracts were used for the measurements of CAT, POD and SOD activities as previously described [32]. The specific activity of GST was assayed in the supernatant by following the increase of absorbance at 340 nm using GST Assay Kit according to the manufacturer instructions (CS0410, Sigma, USA). One unit of activity was defined as the amount of enzyme required to form 1 μM product per minute at 30 °C. Enzyme activities were expressed as U. mg^−1^FW.

### Measurement of GS, GOGAT and Rubisco activities

To prepare the crude enzyme extracts, roots or shoots of each sample were ground into fine powder with liquid N_2_ and homogenized with 50 mM Tris–HCl buffer (pH 7.6, containing 10 mM MgCl_2_, 1 mM EDTA, 1 mM β–mercaptoethanol and 4% (w/v) polyvinylpolypyrrolidone–40) using a chilled pestle and mortar. The homogenate was centrifuged at 15000 g for 30 min at 4 °C and the supernatants were used for the determination of enzyme activities. The glutamine synthetase (GS) activity was measured according to Sakurai’s description [70]. One unit of GS activity was expressed as the amount of enzyme catalyzing the formation of 1 μmol γ-glutamylhydroxamate per min at 37 °C [71]. The glutamate synthase (GOGAT) activity in the supernatants was determined by the conversion of 2-ketoglutarate to glutamate in a reaction mixture containing 200 mM KH_2_PO_4_-KOH pH 7.5, 10 mM glutamine (Gln), 10 mM 2–ketoglutarate, 0.14 mM NADH [72], One unit of GOGAT activity was defined as the oxidation rate of 1 nmol NADH per min at 30 °C. And the Rubisco activity was measured according to the method of Li [73]. One unit of Rubisco activity was defined as the oxidation rate of 1 nmol NADH per min at 25 °C.

### Statistical analysis

Experiment data were expressed as means ± S.E.M. of 3 independent replicates. Statistical differences were evaluated by Duncan’s or t-test with SPSS 13.0 and the level of statistically significant difference was set at p<0.05.

## Supplementary information

**Additional file 1: Figure S1.** H_2_O_2_ localization in situ.

**Additional file 2: Figure S2.** O_2_^−^ localization in situ.

**Additional file 3: Figure S3.** Free amino acid contents assays.

**Additional file 4: Table S1.** Summary of total DEGs identifided in rice roots and shoots following a 4 h rapid NH_4_^+^ accumulation treatment.

**Additional file 5: Table S2.** GO enrichment analysis of DEGsin rice roots and shoots following a 4 h rapid NH_4_^+^ accumulation treatment. (XLS 192 kb)

**Additional file 6: Table S3.** KEGG Enrichment Analysis of DEGsin rice roots and shoots following a 4 h rapid NH_4_^+^ accumulation treatment. (XLS 56 kb)

**Additional file 7: Table S4.** The sequences of primers used for real-time RT-PCR in this research (XLS 27 kb)

## Data Availability

All data supporting the conclusions of this article are provided within the article (and its additional files).

## Abbreviations

AMT: Ammonium transporter
CAT: Catalase
CO_2_: Carbon dioxide
DAB: 3,3′ –diaminobenzidine
DEGs: Differentially Expressed Genes
GABA: Gamma–Aminobutyric acid
GO: Gene Ontology
KEGG: Kyoto Encyclopedia of Genes and Genomes
GOGAT: Glutamate synthase
GS: Glutamine synthetase
GSH: Glutathione
GST: Glutathione S-transferase
H_2_DCF–DA: 2′,7′–Dichlorofluoresceindiacetate
H_2_O_2_: Hydrogen peroxide
LHCs: Light–harvesting complexes
MSX: L–methionine–D,L–sulfoximine
N: Nitrogen
NBT: Nitroblue tetrazolium
NH_4_^+^: Ammonium
qRT-PCR: Quantitative Reverse Transcription Polymerase Chain Reaction
O_2_^−^: Superoxide
POD: Peroxidase
ROS: Reactive Oxygen Species
RNA–Seq: RNA sequencing
SOD: Superoxide dismutase
Rubisco: Ribulose–1,5–bisphosphate carboxylase/oxygenase
TCA cycle: Tricarboxylic acid cycle
