# Supplementary material for: Internal ammonium excess induces ROS-mediated reactions and causes carbon scarcity in rice
Source: BMC Plant Biol. 2020 Apr 7;20:143. doi: 10.1186/s12870-020-02363-x (PMC7140567; doi:10.1186/s12870-020-02363-x)
Supplement: Supplementary file 1 — Additional file 1: Figure S1. H2O2 localization in situ. [file 12870_2020_2363_MOESM1_ESM.doc]

**Figure S1** H_2_O_2_ localization *in situ*. Rice seedlings aged 14 d were subjected to control (1mM NH_4_^+^), high NH_4_^+^ (20 mM) or high NH_4_^+^ + suc (20 mM NH_4_^+^ + 1% sucrose) treatments for 24h. Afterwards, the second leaves or relatively uniform and newly-grown roots of each treatment were stained with 3,3′-diaminobenzidine (DAB). (a) DAB staining of the 2^nd^ leaves. Photos shown leaf segments stained with DAB (+DAB) and the background staining in the absence of DAB (-DAB) was used as negative controls. b) DAB staining of roots and placed with same order as in a). At least three independent samples were used for each experiment. Bars, 200μm.
