# Supplementary material for: Internal ammonium excess induces ROS-mediated reactions and causes carbon scarcity in rice
Source: BMC Plant Biol. 2020 Apr 7;20:143. doi: 10.1186/s12870-020-02363-x (PMC7140567; doi:10.1186/s12870-020-02363-x)
Supplement: Supplementary file 3 — Additional file 3: Figure S3. Free amino acid contents assays. [file 12870_2020_2363_MOESM3_ESM.docx]

**Figure S3** Free amino acid contents assays. Rice seedlings aged 10 d were subjected to a 4h treatment with high NH_4_^+^ (20 mM) in the presence of 1 mM MSX (gray bars). Control was 1 mM NH_4_^+^ (black bars). Values indicated means ± SE of three independent experiments. * and ** represented statistical significances at p ＜ 0.05 and p < 0.01, respectively.
